# Case report: “Proust phenomenon” after right posterior cerebral artery occlusion

**DOI:** 10.3389/fneur.2023.1183265

**Published:** 2023-07-13

**Authors:** Sophie De Beukelaer, A. A. Sokolov, R. M. Müri

**Affiliations:** ^1^Department of Neurology, University Hospital, Inselspital Bern, Bern, Switzerland; ^2^Service de Neuropsychologie et de Neuroréhabilitation, Département des Neurosciences Cliniques, Centre Hospitalier Universitaire Vaudois (CHUV), Lausanne, Switzerland; ^3^Gerontechnology and Rehabilitation Group, ARTORG Center, University of Bern, Bern, Switzerland

**Keywords:** olfaction, limbic system, stroke, medial temporal lobe, hippocampus, emotional regulation

## Abstract

Odors evoking vivid and intensely felt autobiographical memories are known as the “Proust phenomenon,” delineating the particularity of olfaction in being more effective with eliciting emotional memories than other sensory modalities. The phenomenon has been described extensively in healthy participants as well as in patients during pre-epilepsy surgery evaluation after focal stimulation of the amygdalae and post-traumatic stress disorder (PTSD). In this study, we provide the inaugural description of aversive odor-evoked autobiographical memories after stroke in the right hippocampal, parahippocampal, and thalamic nuclei. As potential underlying neural signatures of the phenomenon, we discuss the disinhibition of limbic circuits and impaired communication between the major networks, such as saliency, central executive, and default mode network.

## Introduction

The French literate Marcel Proust, an eponym to the phenomenon, observed that olfactory stimuli are prone to spontaneously elicit vivid and intensively felt autobiographical memories. He meticulously described how the exposure to the smell of a tea-soaked biscuit elicited the sudden rising of a lively childhood memory closely linked to a feeling of intense joy (1). Research has investigated the capacity of olfactory stimuli to trigger emotion and memory effectively. Indeed, most studies in humans, although few in number, pointed at the capacity of odors to serve as significant context cues underlying the formation and later retrieval of content-dependent odor-evoked autobiographical memories (2, 3). Furthermore, odor-evoked memories were found to be particularly emotional as measured by both self-report and heightened heart rate responses, especially in comparison to memories elicited by other modalities (visual, tactile, and auditory) (1, 4–6). These characteristics have been related to the specific connectivity between olfactory and limbic structures, permitting the integration of olfactory information and mnemonic processes in a way that affective, visceral, and motor responses to odors can be generated congruously (3, 7–9). The neural structures implied in olfactory processing are the primary olfactory (piriform) cortex, the amygdalae, the hippocampus and the surrounding cortex, the anterior insulae, the orbitofrontal cortices, and parts of the medial thalamus. In contrast to other sensory information, olfactory signals have a dual route to the neocortex: one relaying to parts of the medial thalami nuclei and one projecting directly from the bulbs to the primary olfactory cortices and limbic structures (2, 3, 10, 11).

Direct cortical stimulation during pre-surgical exploration in patients suffering from epilepsy offered more insight into the potential neural underpinnings of the “Proust phenomenon,” reporting the induction of the phenomenon via focal electrical stimulation of the amygdalae. Indeed, the stimulation of the basolateral nuclei of the left amygdala in a patient led to the sudden reminiscence of a smell of burnt wood evoking a campfire scene associated with the feeling of intense joy. The patient herself did not suffer from olfactory hallucination or déjà-vu before the stimulation, the semiology of her seizures being complex motor semiology since the age of 5, following a small lesion of the left frontal lobe (10, 12). Furthermore, a study of bilateral amygdala stimulation evinced that both positive and negative valence could be linked to a scenic memory when stimulating the left amygdala, while stimulation of the right amygdala resulted only in negatively valenced reminiscence (13, 14). A divergence is conjectured to be the result of asymmetrical processing of visceral signals in the insular cortices, which are structures with which the amygdalae are known to interact closely (2, 12, 15, 16).

Furthermore, the effectiveness of olfactory stimuli in triggering emotive memories becomes relevant in patients suffering from post-traumatic stress disorder (PTSD), especially of the non-dissociative type, where odor-evoked aversive autobiographical memories are a particularly debilitating issue. The neural structures facilitating olfactory processing overlap with those known to have altered functionality in patients with PTSD, where a threat triggered unexpectedly by an odor may be discrepant with the actual danger of the present situation and results in involuntary intrusions and flashbacks (2, 3, 17). Even healthy veterans display changes in processing the intensity of aversiveness related to contextually relevant odors, such as diesel or rubber (18), suggesting that the pairing among odor, arousal, and valence in becoming a relevant threat may be gradual, may even be time-limited, and possibly responsive to context-dependent learning *via* interoceptive and exteroceptive cues (19).

To the best of our knowledge, the appearance of a “Proust phenomenon” has not yet been described following a stroke.

## Case

**Figure 1:**
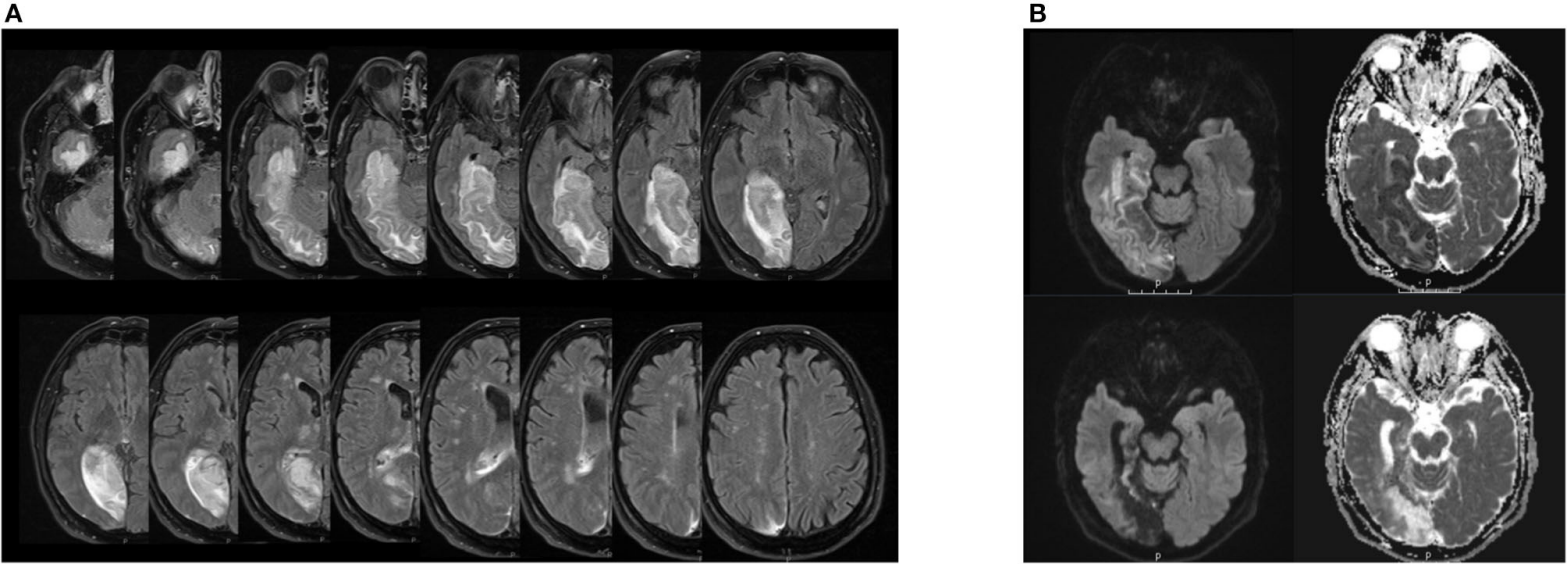
**(A)** Lesion extension in patient EP in the follow-up MRI after acute treatment. Right hemisphere is on the left side of the each image respectively. FLAIR sequence from a cerebral magnetic resonance imaging, 3 Tesla, with on the upper left side of the image the inferior and the lower side of the image the upper bound of lesion estension. The hyperintense signal displays the demarcated subacute ischemic and hemorrhagic transformations in the right PCA-territory encompassing the right thalamus, cuneus, hippocampus, gyrus parahippocampalis and the precuneus as well as the right medial and posterolateral thalamus. **(B)** Hippocampal lesion in patient EP in the follow-up MRI after acute treatment (upper images) and 3 months after symptom onset (lower images). Right hemisphere is on the left side of each image respectively. DWI sequence from a cerebral magnetic resonance imaging, 3 Tesla, on the left side b 1,000 and the ride side the ADC map. On the upper images, the diffusion restriction encompasses the hippocampus, the gyrus parahippcampalis and the temporo-occipital lobes depicted. Note the cortical diffusion restriction of the vental hippocampus. On the lower pictures 3 months after symptom onset, we find the post-ischemic parenchymal lesion of the formerly diffusion restricted regions with evacuo configurartion of the temporal ventricle and loco regional subarachnoid space.

A 64-year-old male patient was presented to the emergency ward with left-sided hemiparesis, sensory hemisyndrome, homonymous hemianopia, as well as left spatial neglect. The initial cerebral MRI showed an ischemic lesion in the territory of the right posterior cerebral artery (PCA) with occlusion of the P2-segment. Areas affected were the right gyrus parahippocampalis, right hippocampus, and posterior-medial thalamus without signs of hemorrhage. Endovascular treatment was administered including intra-arterial lysis, leading to complete recanalization of the right PCA (TICI 3). The follow-up MRI showed demarcated subacute ischemic lesions in the PCA territory, complicated by the hemorrhagic transformation of the right medial and posterolateral thalamus, right cuneus, precuneus, complete hippocampus, and gyrus parahippocampalis (see Figure 1). Stroke etiology was considered cardiac with a permeable foramen ovale as seen in the trans-esophageal echocardiography and a risk of paradoxical embolism (RoPE score) of 5, in the absence of other causes. The patient had been absolved from primary school and then worked as a farmer. His wife died 6 years before his stroke. An intensive inpatient neurorehabilitation with physical, occupational, speech therapy, and neuropsychology was initiated on the 7th-day post-stroke.

During the clinical examination at the neurorehabilitation onset, the patient showed a left sensorimotor hemisyndrome with dysesthesia, homonymous hemianopia, severe topographagnosia, and deficits in visuo-construction, as well as deficits in the executive domain. Furthermore, the patient reported the sudden upcoming of a scenic childhood memory including rotten piglets at the farm he grew up on when smelling the odor of meaty dishes, associated with a feeling of strong repulsion and disgust. That odor-evoked autobiographical memory was unknown before the stroke but only triggered when smelling meaty substances and was always reported as highly aversive. However, the patient could taste, smell, and distinguish properly different odors on Sniff tests,^1^ a clinical olfactory assessment. He had no psychiatric or neurological condition before the stroke onset, no history of drug abuse, long-term medical treatment, no current psychosocial stress factors, and no pronounced commitment to vegetarianism or veganism. Furthermore, the reminiscence was context-dependent and never occurred without the specific olfactory stimulus. These odor-evoked memories impaired the patient considerably, leading to a reduced appetite, weight loss of ~13 pounds, and an important psychological strain. The severity of the “Proust phenomenon” regressed moderately under therapy with selective serotonin reuptake inhibitors as well as pregabalin, food counseling, and neuropsychological therapy. After discharge, aversive memories could still be triggered by the smell of meaty substances but were not experienced as debilitating.

## Discussion

Our patient started presenting the “Proust phenomenon” after the stroke to the right PCA territory. He never witnessed intrusions or odor-triggered memories before the incident. Hence, we suggest that stroke lesions must have disrupted the network integrity and communication of olfactory limbic circuitry resulting in the attribution of negatively valenced salience to formerly neutral olfactory stimuli. In particular, we think that lesions in the right hippocampal structures may have disrupted the functional integrity at the circuit and network level, thereby facilitating signal processing in olfactory limbic circuits.

In our patient, the complete right hippocampus was damaged (see Figure 1B). Anterior and posterior subregions of the hippocampus have distinct functional and structural connections, with the anterior hippocampus being active when the context is evaluated and the posterior hippocampus when precise spatial location is assessed (20). Furthermore, the anterior hippocampus, structurally linked to the amygdalae, the limbic prefrontal areas, and the hypothalamic-pituitary axis, has been shown to be active in emotional memory (21) and reward-directed behavior (22). The posterior hippocampus is connected with cingulate areas, notably the anterior cingulate cortex (ACC), posterior cingulate cortex (PCC), and precuneus (23), and contributes to the default mode network (DMN). Importantly, the amygdalae and their projections to the orbitofrontal and insular cortices, structures of the central olfactory matrix, were preserved in our patient. Thus, direct and bilateral processing of olfactory stimuli by the amygdalae and further cortical projections remained intact.

We propose that the lesion of the right anterior hippocampus in our patient and its consecutive hypoactivation may have disrupted the connectivity between the anterior hippocampus and the amygdala, facilitating the disinhibition of olfactory-limbic processing and associative learning mechanisms driven among others by the amygdalo-insular pathways. Hypoactivation may have ensued in a lesser ability to disambiguate the context when exposed to the smell of meat, henceforth triggering repulsive childhood memories, the clinical presentation of the “Proust phenomenon.”

Earlier research in PTSD highlights the importance of the hippocampus–amygdala circuit for further emotion regulation in autobiographic memories and lends support for our interpretation. Patients with PTSD without dissociative symptoms may re-experience events vividly through salient olfactory stimuli (6). Studies suggest that those patients are not necessarily displaying a globally enhanced fear expression when exposed to salient stimuli but rather an impaired capacity to use contextual information to disambiguate potential safety and threat. As we previously highlighted, the process of disambiguating stimuli is known to be a hippocampus and parahippocampus-dependent process. Indeed, Garfinkel showed that PTSD patients were less effectively using the safety context to suppress fear memory than a danger context to enhance it (19). Furthermore, studies revealed that in addition to displaying reduced hippocampal neuronal integrity, patients with PTSD showed increased amygdala activity, suggesting enhanced fear signal processing (19). Taken together, we postulate a meaningful neurobiological similarity between the disinhibited amygdala-insular pathways due to anterior hippocampal lesion in our patient compared to the hippocampal under activity and amygdalae hyperactivity in patients with PTSD and non-dissociative symptoms when exposed to salient olfactory stimuli, leading to the “Proust phenomenon.”

Furthermore, the negative valence of the emotion associated with the triggered autobiographical memory in our patient is not arbitrary as earlier research in the “Proust phenomenon” has shown that hemispheric laterality mattered for the valence attributed to the evoked autobiographical memory after stimulation of the amygdalae, with right amygdala stimulation ensuing in negatively evoked memories and left amygdala stimulation in both positively and negatively valenced evoked memories (10, 13, 15). As smell has been linked to precipitating fear- and anxiety-related memories in PTSD patients without dissociative symptoms, it is worthwhile highlighting here that deficits in intrinsic sensory inhibition have just recently been found to contribute to intrusive trauma re-experiencing mediated by olfactory memory (17). Additionally, the olfactory cortex and its extended circuit encompass the amygdala, hippocampus, orbitofrontal cortex, insula, and the ACC, which receive a significant bottom–up input from the primary olfactory cortex when triggered by an odor (3), areas left untouched by lesions in our patient. Although neural communication in patients with PTSD is still poorly understood, especially in the domain of olfaction, studies found increased functional connectivity between the right anterior insula and amygdala among PTSD patients at rest (24) as well as when repeatedly exposed to traumatic memory (25). Furthermore, particularly the anterior insula, the ACC, and their functional coupling within the salience network are active in the evaluation of disgust and consecutive avoidance behavioral (15, 26) symptoms that our patient witnessed when exposed to the smell of meaty substances.

Interestingly, facilitated olfactory-limbic processing results in a heightened activity of regions that constitute key components of the distributed salience network. This network, including the bilateral anterior insulae, ACC, amygdalae, and hippocampi, is involved in the detection of homeostatically relevant inputs, i.e., the detection, learning, and modulation of salient events and the promotion of appropriate behavioral adjustment with strong functional coupling to the motor system (15, 27). Furthermore, the salience network is thought to arbitrate the functional dynamics between the central executive network (CEN) and DMN (28). In patients with PTSD without dissociative symptoms, functional neuroimaging studies indicated an at-rest overactivity and hyper-connectivity of the salience network eventually resulting in a low threshold for perceived salience, facilitated bottom-up processing, and an inefficient DMN-CEN modulation (17, 29, 30). Indeed, our patient's repeated and effective olfactory-induced, repulsive memories share many features clinically with the olfactory intrusions reported by patients suffering from PTSD without dissociative symptoms most likely facilitated by the overactivity and hyper-connectivity of the salience network.

Notably, we observed that the lesions of higher visual areas, the visual tract, and the precuneus may have complicated stimulus reappraisal in our patient as exteroceptive updating through visual pathways of relevant olfactory cues (19) was less available. Indeed, research in healthy participants showed that an intact amygdala–precuneus connectivity correlated positively with eye-tracking measures of successful attentional deployment suggesting that diverting attention away from arousing information depends on the functional relationship between limbic and parietal structures, essential for emotion regulation strategies (31, 32).

Finally, preserved amygdala integrity allowed the ultimately efficacious behavioral therapy approach in our patient with reconditioning the formerly neutral stimulus of a meaty dish (32, 33).

## Conclusion

While perceptual disorders often develop after stroke (34), we here report for the first time a “Proust phenomenon” following a right posterior cerebral artery stroke. The putative underlying mechanisms encompass aberrant processing in olfactory-limbic networks and their deficient integration with larger neural ensembles, such as the salience and default mode networks. In our patient, we hypothesize deficient contextualization of the olfactory stimulus due to lesions in the right anterior hippocampus, thereby disinhibiting negatively valenced olfactory limbic processing and associative learning mechanisms. Understanding the circuit- and network-level structural and effective connectivity (35, 36) of neurocognitive and behavioral phenomena is crucial as it enables the development of efficacious and individualized treatment options in neurorehabilitation. Close interaction and interdisciplinary interpretation of the observed phenomena in neurology and psychiatry also appear indispensable as the behavior often relies on similar or overlapping circuits (37, 38).

## Data availability statement

The original contributions presented in the study are included in the article/supplementary material, further inquiries can be directed to the corresponding author.

## Ethics statement

Ethical review and approval was not required for the study on human participants in accordance with the local legislation and institutional requirements. The patients/participants provided their written informed consent to participate in this study. Written informed consent was obtained from the individual(s) for the publication of any potentially identifiable images or data included in this article.

## Author contributions

SB wrote the manuscript. AS and RM commented and proof-corrected it. All authors contributed to the article and approved the submitted version.

## Footnotes

^1^Screening test for Anosmia. See: Kobal G, Hummel T, Sekinger B, Barz S, Roscher S, Wolf S. “Sniffin' Sticks”: screening of olfactory performance. Rhinology 1996; 34:222-226; Wolfensberger M, Schnieper I, Welge-Lüssen A. Sniffin' Sticks: a new olfactory test battery. Acta Otolaryngol 2000;120:303-306.
